# A Case of Hydatids of the Lungs

**Published:** 1882-11

**Authors:** D. F. Smith


### A CASE OF HYDATIDS OF THE LUNG.

Miss K., a rather dark, muddy-complexioned girl, æt. 20 years, came to my office on November 4th, 1881, to consult about a runniug sore on the back of her chest. It was situated within two inches of the spine, and opposite the 8th intercostal space of the left side. There was a good deal of flabby enlargement in the neighborhood of this spot, and by probing, several sinuses were found running upward and outward from the common outlet or sore. There was no other bulging upon the chest; she had no cough, pain, or shortness of breath; but she was weak, emaciated, and had a poor appetire ; catamenia normal, and no alteration in the pupils or superficial veins of thorax ; liver and kidneys healthy. She gave the following history :

About two years previous, complained for the first time of ill-health, which developed from a pain in the side and situation above-mentioned, soon followed by slight shortness of breath and very little cough ; in fact, she scarcely remembered having had a cough till I questioned her upon that point ; no expectoration. These symptoms, which were so slight that they were never considered in connection with her trouble, were also accompanied by occasional chills, fever and sweating, and continued about six months, with considerable loss of flesh and reduced strength, when a tumour as large as the closed fist developed near the inferior angle of the scapula. It had been diagnosed and treated as fatty tumour, cold abscess, &c., on two or three occasions, but pus invariably escaped on applying the lancet. Nothing extraordinary had been noticed about the pus, but the opening never completly closed, while matter always reaccumulated and formed a fresh tumour in about the same locality. In this way three successive abscesses was formed and their contents discharged. The patient objected to my laying open the sinuses, as I advised, and went away with a tonic and a solution of iodine with which to syringe the channels leading from the sore. On the 30th of November the patient was no better, but had decided to let me proceed as I thought best. She was accordingly chloroformed and the sinuses laid open by an incision extending from the 6th to the 9th ribs, and two inches to the left of the spine. The probe could now be made to pass deeply through three small openings in the 6th, 7th and 8th intercostal spaces. Two transverse slits an inch and a half long, corresponding with the highest and lowest openings between the ribs, were then made (upon a director) to connect with the first incision, when a considerable quantity of dirty greyish-brown-looking pus escaped through the new-made openings. The finger could now be easily passed through either intercostal slit, by which the costal walls of the cavity were felt smooth, regular and unyielding. The finger came in contact with no resisting substance in any other direction, except slight aortic pulsation. By means of an injection (iodised) made through fthe upper opening, pus and a number of different sized small, smooth and slippery walled cysts were forced through the lower opening, till the uncolored injection and exploration with the finger indicated that the cavity was well cleaned out. The cysts were all open when they escaped, and quite collapsed. There would be none larger than a large-sized marble if distended to their fullest extent. Some of the cysts still contained a small quantity of slimy, semi-transparent glairy fluid. During the subsequent treatment, an occasional sac of the same character came away with the process of syringing. No microscopic examination of the contents of the cavity was made. The after-treatment consisted in washing out the cavity every day with injections of tr. iodine and water, 1 to 4, and sometimes 1 to 2, with the internal administration of iron and quinine. Drainage tubes were employed all through the treatment. The case progressed well, and recovery was complete in two months.

I have only seen the case two or three times since, but noticed that, although fairly well nourished, she had a cachectic appearance, but no cough, pain, or shortness of breath. She is at present teaching school.—D. F. SMITH, *in Canada Med. and Surg. Jour.*

